# Maternal High Fat Diet and in-Utero Metformin Exposure Significantly Impact upon the Fetal Renal Proteome of Male Mice

**DOI:** 10.3390/jcm8050663

**Published:** 2019-05-11

**Authors:** Eva Nüsken, Eva-Maria Turnwald, Gregor Fink, Jenny Voggel, Christopher Yosy, Tobias Kretschmer, Marion Handwerk, Maria Wohlfarth, Lutz T. Weber, Eva Hucklenbruch-Rother, Jörg Dötsch, Kai-Dietrich Nüsken, Sarah Appel

**Affiliations:** Department of Pediatrics and Adolescent Medicine, University of Cologne, Medical Faculty and University Hospital Cologne, 50937 Cologne, Germany; eva-maria.turnwald@uk-koeln.de (E.-M.T.); gregor.fink@uk-koeln.de (G.F.); jenny.voggel@uk-koeln.de (J.V.); christopher.yosy@gmx.de (C.Y.); tobias.kretschmer@uk-koeln.de (T.K.); marion.handwerk@uk-koeln.de (M.H.); maria.wohlfarth@uk-koeln.de (M.W.); lutz.weber@uk-koeln.de (L.T.W.); eva.rother@uni-koeln.de (E.H.-R.); joerg.doetsch@uk-koeln.de (J.D.); kai-dietrich.nuesken@uk-koeln.de (K.-D.N.); sarah.appel@uk-koeln.de (S.A.)

**Keywords:** fetal development, high fat diet, intrauterine programming, kidney, metformin, maternal obesity

## Abstract

There is accumulating evidence for fetal programming of later kidney disease by maternal obesity or associated conditions. We performed a hypothesis-generating study to identify potentially underlying mechanisms. Female mice were randomly split in two groups and fed either a standard diet (SD) or high fat diet (HFD) from weaning until mating and during pregnancy. Half of the dams from both groups were treated with metformin ((M), 380 mg/kg), resulting in four experimental groups (SD, SD-M, HFD, HFD-M). Caesarean section was performed on gestational day 18.5. Fetal kidney tissue was isolated from cryo-slices using laser microdissection methods and a proteomic screen was performed. For single proteins, a fold change ≥1.5 and *q*-value <0.05 were considered to be statistically significant. Interestingly, HFD versus SD had a larger effect on the proteome of fetal kidneys (56 proteins affected; interaction clusters shown for proteins concerning transcription/translation, mitochondrial processes, eicosanoid metabolism, H2S-synthesis and membrane remodeling) than metformin exposure in either SD (29 proteins affected; clusters shown for proteins involved in transcription/translation) or HFD (6 proteins affected; no cluster). By further analysis, ATP6V1G1, THY1, PRKCA and NDUFB3 were identified as the most promising candidates potentially mediating reprogramming effects of metformin in a maternal high fat diet.

## 1. Introduction

Pregnancy disorders not only threaten the well-being of mother and child but also impair the normal development of the fetus. Fetal kidney development significantly depends upon sufficient supply of macro- and micronutrients and can be adversely affected by maternal malnutrition, placental dysfunction, gestational diabetes, prematurity and intrauterine stress [1]. Amongst these risk factors, maternal obesity is probably the most prevalent condition worldwide. Epidemiologic studies from the USA, England and Australia reported that about 20–30 percent of pregnant women were overweight and up to 20 percent were obese [2,3,4]. These women are at higher risk to develop gestational diabetes, arterial hypertension and preeclampsia [5,6]. Consequently, a significant proportion of the present pediatric population may have experienced adverse renal programming, leading to arterial hypertension and chronic renal disease during later life [1,7].

Metformin has increasingly been used to treat gestational diabetes during recent years [8,9] and no major sequelae for the unborn child have been reported [10,11,12]. However, metformin is known to pass the placenta [13,14]. Data on long-term outcomes of children exposed to metformin regarding fetal programming events in utero are scarce. First studies in children born to mothers treated with metformin due to gestational diabetes mellitus hint at an accelerated growth in early childhood compared to children whose mothers were treated with insulin. These data support the hypothesis that in utero exposure to metformin might have programming effects. Psychosocial development was not different between the two groups [15]. Similarly, blood pressure levels at two years of age were comparable between children born to diabetic mothers treated with metformin and diabetic mothers treated with insulin [16]. Long-term renal outcomes in these children haves not been studied so far.

Since prevention of chronic kidney disease remains a major public health challenge [17] and renal programming contributes considerably to susceptibility towards chronic renal disease [1], further understanding of the exact mechanisms leading to renal programming in maternal obesity is highly warranted. 

In our laboratory, a high fat diet before and during pregnancy is an established model to study maternal obesity in mice [18,19,20,21]. In this study, the experimental set-up was designed to analyze both the effect of maternal high fat diet and the effect of metformin exposure with or without the background of maternal high fat diet on the renal proteome of fetal male mice, and to use these data to generate new hypotheses about which proteins may be involved in fetal kidney programming under a high fat diet and metformin exposure. 

## 2. Materials and Methods

### 2.1. Animal Model

All procedures were conducted in accordance with the German regulations and legal requirements. The experimental protocol was approved by the appropriate Institutional and Governmental Review Boards.

Mice (C57BL/6N; Janvier Labs, Le Genest-Saint-Isle, France) were bred locally at a designated animal unit of the University Hospital of Cologne (Cologne, Germany). All animals were housed in individually ventilated cages (IVCs Blue Line, Tecniplast, Buguggiate, Italy) providing a temperature of 22 °C ± 2 °C; constant relative humidity of 50–60% and were kept under a 12-hour light, 12-hour dark cycle. After weaning, female mice were fed ad libitum either a standard diet (SD; R/M-H ssniff®, Soest, Germany) containing 9% fat of metabolizable energy or a high fat diet (HFD; C1057 mod, Altromin, Lage, Germany) containing 60% fat of metabolizable energy (for details please see [21]). At 12–16 weeks of age, SD and HFD females were mated overnight and the next day randomized to either receive metformin via drinking water (380 mg/kg body weight/day) throughout pregnancy (groups SD-M, HFD-M) or no treatment (groups SD, HFD). The type of diet was not changed during pregnancy. The day after mating was considered as gestational day (GD) 0.5. Shortly before term, on GD 18.5, dams were anesthetized with buprenorphine (0.1 mg/kg body weight; s.c. injection) and underwent a cesarean section. After delivery, fetuses were weighed and sacrificed by decapitation. Half of the fetuses were then placed into Tissue-Tek® O.C.T.™ Compound (Sakura, Alphen aan den Rijn, Netherlands) and cryoconserved at −80 °C. Residual fetuses were either used for single organ harvesting or were fixated in formalin for further immunohistochemical analyses. In this study, only litters with at least 5 viable pups were included in the analyses. All body weight values in the text are mean ± SD.

### 2.2. Sex-PCR

After dissection and weighing, a tail cut was performed in order to determine the sex of every fetus. DNA was extracted by lysing the tissue in lysis buffer (100 mM Tris, 5 mM EDTA, 200 mM NaCl, 0.2% SDS, pH 8.0) supplemented with 3–5 units Proteinase K (Thermo Scientific, Waltham, MA, USA) over night at 55 °C. The next day, DNA was precipitated with isopropanol, spun down and the DNA-pellet was washed with 70% ethanol. DNA was dissolved in water and used as the template in a PCR reaction. Briefly, 1 µL DNA was mixed with 9 µL PCR-mix (1× GoTaq Flexi buffer (Promega, Madison, USA), 3 mM MgCl2, 0.2 mM dNTPs, 0.4 µM of each primer, 0.5 units GoTaq (Promega, Madison, USA) and fragments of the X (104 bp) and Y-chromosome (344 bp) were amplified (95 °C for 3 min, 35 cycles of: 95 °C for 30 seconds, 58 °C for 1 min, 72 °C for 1 min, following a final elongation step of 72 °C for 10 min). The following primer pairs were used: Y-chromosome: Y-for 5′-CTGGAGCTCTACAGTGATGA-3′, Y-rev 5′-CAGTTACCAATCAACACATCA-3′, X-chromosome: X-for 5′-AACATCCTGAACACCTTGCC-3′, X-rev 5′-TAGCTTGTGGCTCTCCAGGT-3.

### 2.3. LCM Procedure and Protein Extraction

For this study, only male embryos were used (*n* = 5 embryos in groups SD-M, HFD and HFD-M, *n* = 3 embryos in group SD). Embryos were sectioned on a Cryostat (Leica CM3050 S; Leica, Wetzlar, Germany) at −21 °C to 16 µm thick sections. Consecutive sections were collected onto PEN-Membrane Slides (Leica No. 11600288; Leica, Wetzlar, Germany) and placed on dry ice for transfer. A standard hematoxylin and eosin staining was applied to discriminate between organs and to localize both kidneys. Briefly, sections were fixed in 4 °C 70% ethanol combined with a cOmplete® protease inhibitor cocktail (Roche, Basel, Switzerland) for 1 min, rinsed in double-distilled water (ddH_2_O), stained in Mayer’s hematoxylin for 1 min., dipped in eosin, rinsed in 70% ethanol, and left to dry at room temperature for at least 1 hour before laser capture microdissection was performed.

**Figure 1:**
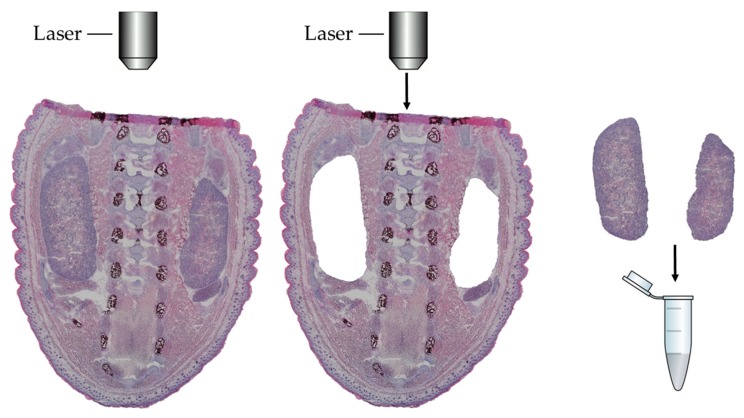
Representative example of laser dissection is shown.

A slide with air-dried sections was inserted in the slide holder of the LCM microscope (LMD7000, Leica Microsystems; Leica, Wetzlar, Germany; power 16, aperture 20, velocity 7), and a 200 µL PCR-tube was placed in such a way underneath the sections that dissected pieces fell directly into the tube cap. Approximately 12 mm^2^ of kidney were collected per embryo (Figure 1). Consecutively, the tubes were removed from the holder, and the contents were carefully centrifuged to the bottom of the tube before 50 µL of 10% SDS in PBS buffer was added. Tubes were stored at −20 °C for one night before the protein was extracted.

Proteins were extracted and processed using an approach for LCM samples containing minimal amounts of protein in the (sub-) microgram range). For this purpose, samples were heated to 95 °C for 10 min followed by chromatin degradation using a Bioruptor for 10 min, cycle 30/30 seconds. Subsequently, DTT was added to a final concentration of 5 mM, vortexed, and incubated for 30 min at 55 °C. Next, CAA was added to a final concentration of 40 mM, vortexed, and incubated in the dark for 30 min at room temperature. Proteins were digested with trypsin, and peptides were purified and stored at −20 °C prior LC-MS analysis.

### 2.4. Mass Spectrometry [as Performed by the Proteomics Facility of the Cluster of Excellence—Cellular Stress Responses in Aging-Associated Diseases (CECAD) Cologne]

A Q Exactive Plus Orbitrap (Thermo Scientific, Waltham, MA, USA) mass spectrometer coupled to an EASY nLC 1000 (Thermo Scientific, Waltham, MA, USA) was used to analyze all samples. Loading of peptides was done with solvent A (0.1% formic acid in water) onto an analytical column (50 cm—75 µm I.D., filled with 2.7 µm Poroshell EC120 C18, Agilent, Santa Clara, CA, USA) packed in-house. Chromatographical separation of peptides was performed at a constant flow rate of 250 nL/min using the following gradient: 3–5% solvent B (0.1% formic acid in 80% acetonitrile) within 1.0 min, 5–30% solvent B within 119.0 min, 30–50% solvent B within 19.0 min, 50–95% solvent B within 1.0 min, followed by washing and column equilibration. The data-dependent acquisition mode was used on the mass spectrometer. The MS1 survey scan was acquired from 300–1750 m/z at a resolution of 70,000. Isolation of the top 10 most abundant peptides was performed within a 2.1 Th window and peptides were subjected to HCD fragmentation at a normalized collision energy of 27%. A maximum injection time of 60 milliseconds was applied, as the AGC target was set to 5e5 charges. Product ions were detected at a resolution of 17,500 in the Orbitrap. Dynamic exclusion of precursors was set for 25.0 seconds.

### 2.5. Proteomics Data Analysis (as Performed by the Proteomics Facility of the CECAD Cologne)

Proteomics data analysis was performed using free academic software Maxquant (https://maxquant.org/maxquant/, version 1.5.3.8) with default parameters to process all mass spectrometric raw data. In short, MS2 spectra were searched against the Uniprot MOUSE.fasta database, with a list of common contaminants included. False discovery rates (FDR) on protein and peptide spectrum matches (PSM) level were estimated by the target-decoy approach to 1% (Protein FDR) and 1% (PSM FDR), respectively. The minimal peptide length was set to 7 amino acids and carbamidomethylation at cysteine residues was considered as a fixed modification. Oxidation (M) and Acetyl (Protein N-term) were included as variable modifications. The match-between runs option was enabled. Label-free quantification (LFQ) was enabled using default settings. 

**Figure 2:**
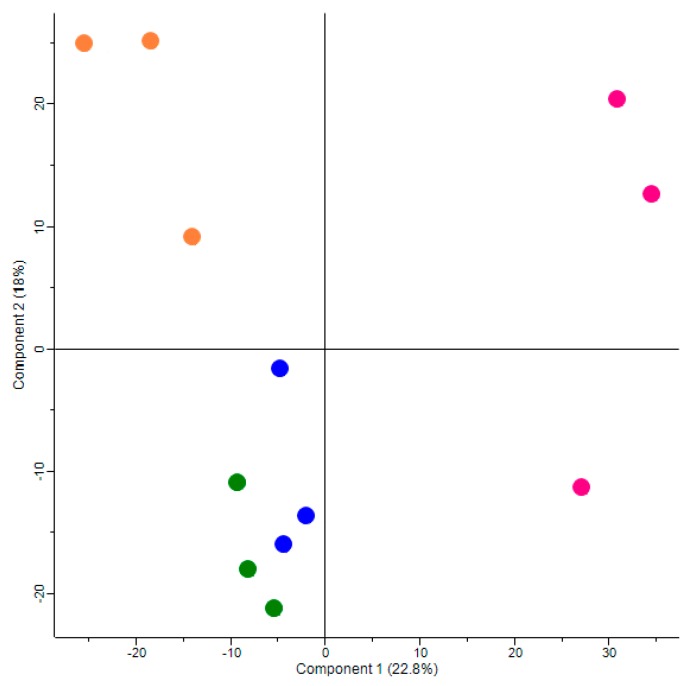
Principal component analysis. Orange dots, HFD group; pink dots, SD group; blue dots, HFD-M group; green dots, SD-M group.

A principal component analysis (PCA) was performed to analyze clustering of individual samples within the respective experimental groups and to identify outliers. In all experimental groups, *n* = 3 animals per group could be clustered by PCA analysis (Figure 2). These samples were used for further analysis. 

Further analysis was carried out using free software Perseus (https://maxquant.org/perseus, version 1.5.5.3). Briefly, LFQ values were log2 transformed, and proteins flagged as “potential contaminants”, “reverse” and “only identified by site” were removed from the data set. Three group comparisons of interest were defined as follows: (1) HFD vs. SD, (2) SD-M vs. SD, (3) HFD-M vs. HFD. For each protein a two-sample *t*-test was used to determine statistically significant changes (*p* < 0.05). To reduce the number of false positive findings amongst the list of proteins due to the large number of single *t*-tests, *p*-values were corrected for multiple testing by using the Benjamini–Hochberg correction. The resulting *q*-values were used for identification of significantly altered proteins (*q* < 0.05). 

### 2.6. Further Data Processing

**Figure 3:**
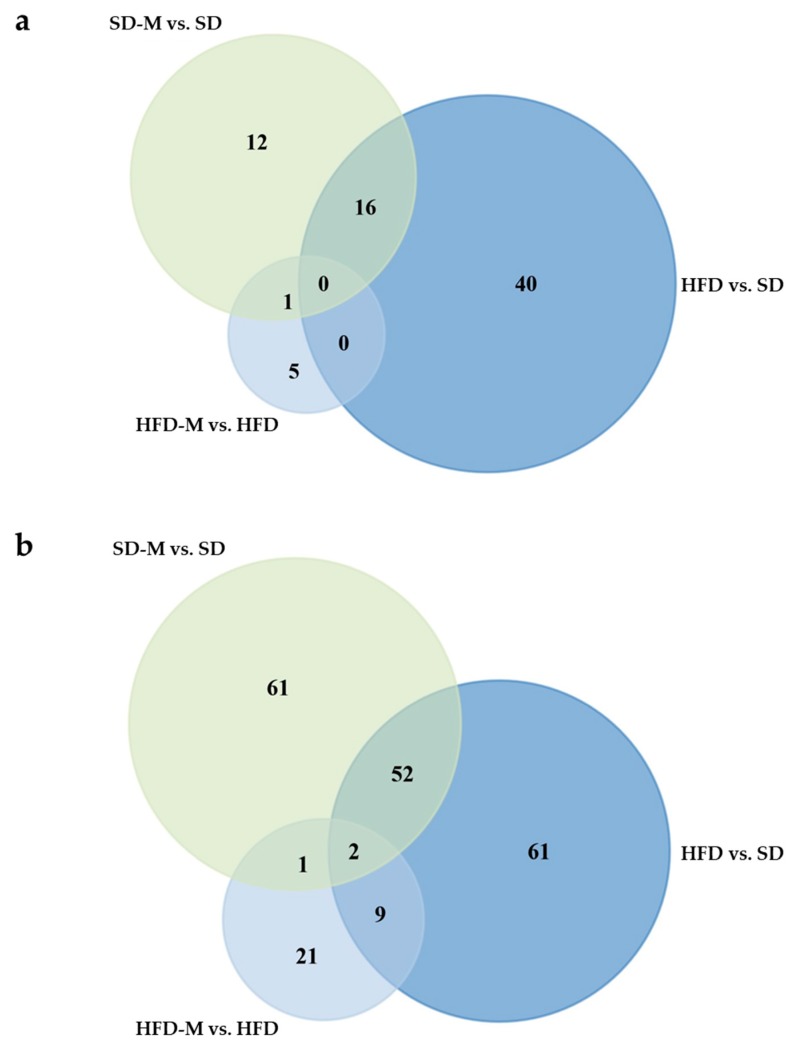
(**a**) Venn diagram to visualize number of overlaps of significantly (*q* < 0.05) and relevantly (fold change ≥1.5) altered proteins of the comparisons SD-M vs. SD (i.e., number of renal proteins altered by metformin in fetuses of dams fed a standard diet), HFD vs. SD (i.e., number of renal proteins altered by high fat diet) and HFD-M vs. HFD (i.e., number of renal proteins altered by metformin in fetuses of dams fed a high fat diet); (**b**) Venn diagram to visualize number of overlaps of considerably (*q* < 0.1) and relevantly (fold change ≥1.5) altered proteins of the same comparisons.

Subsequently, we used String database analysis (www.string-db.com, version 11.0) to identify molecular interactions (based on evidence from experiments or curated databases) and functional enrichments concerning biological processes or Uniprot Keywords (false discovery rate FDR <0.01) of all significantly (*q* < 0.05) and relevantly (fold change, fc ≥ 1.5) altered proteins. For the identification of altered and overlapping proteins between single groups or group comparisons, an overlap analysis (Venn diagram) of all significantly (*q* < 0.05) and relevantly (fc ≥ 1.5) altered proteins of the comparisons HFD vs. SD, SD-M vs. SD and HFD-M vs. HFD was generated (Figure 3a) using open access software FunRich (http://funrich.org, version 3.1.3). Additionally, a second overlap analysis was performed of the same comparisons using a *q*-value of <0.1 and an fc ≥ 1.5 in order not to miss considerable effects by the strict *q*-value cut-off of <0.05, especially focusing on metformin effects (Figure 3b).

## 3. Results

### 3.1. Animal Phenotype

At the time of mating, female lean mice fed a standard diet (SD) displayed a body weight (21.9 ± 1.1 g) significantly lower compared to the body weight of obese (HFD) dams (25.3 ± 1.9 g) (*p*-value < 0.001; Mann Whitney test). As known from earlier studies, HFD mice fasting blood glucose levels are elevated and glucose tolerance is disturbed compared to SD mice [19]. So far, the metformin treated dams (SD-M and HFD-M groups) still need to be characterized in regard to glucose metabolism, and experiments are on the way. The fetal phenotype of SD and HFD mice has been described earlier, showing lower body weight of fetuses from dams fed HFD compared to dams fed SD at gestational day (GD) 15.5, followed by an intrauterine catch-up growth within the next 3 days resulting in similar body weight of fetuses from both experimental groups at GD 18.5 [21]. As expected, in this study also fetuses at GD 18.5 displayed body weights not significantly different when comparing SD (1.10 ± 0.12 g) and HFD (1.08 ± 0.16 g) mice. Treatment with metformin during pregnancy did not significantly change fetal body weight at GD 18.5, either in SD (SD-M: 1.17 ± 0.19 g) or in HFD (HFD-M: 1.15 ± 0.14 g) fetuses. Moreover, the resorption rate (equivalent to miscarriage in humans) and litter size did not differ due to HFD feeding or metformin treatment. 

### 3.2. Principal Component Analysis Iindicates a Strong and Differential Effect of Maternal High Fat Diet and Metformin Treatment on Global Protein Expression in Fetal Kidneys 

Principal component analysis (Figure 2) indicated that the global protein expression patterns both in fetuses of dams fed a high fat diet without metformin (HFD group) and in fetuses of dams fed a standard diet without metformin (SD group), clearly differed compared to each other and compared to both the groups fed additional metformin throughout pregnancy. Interestingly, there was a common cluster of fetuses of dams fed a high fat diet with metformin (group HFD-M) and fetuses of dams fed a standard diet with metformin (group SD-M), suggesting a major effect of metformin exposure on global protein expression regardless of diet before and during pregnancy. 

### 3.3. Overlap Analyses Identify 16 Proteins Which Were Similarly Altered by High Fat Diet and Metformin During Standard Diet, and 11 Proteins Which Were Dysregulated by High Fat diet and Counter-Regulated by Metformin 

Fifty-six proteins were significantly (*q*-value < 0.05) and relevantly (fold change ≥1.5-fold) altered in fetuses by a high fat diet (Table 1; comparison of HFD group to SD group), 29 proteins by metformin in fetuses fed a standard diet (Table 2; comparison of SD-M group to SD group), and 6 proteins by metformin in fetuses fed a high fat diet (Table 3; comparison of HFD-M group to HFD group). Interestingly, the first overlap analysis identified 16 proteins which were similarly altered by HFD and by metformin during SD (Figure 3a shows Venn diagram, Table S1 shows overlapping proteins). String analysis even identified an interaction of three of the overlapping proteins (Figure S1). 

However, we did not identify many similar effects of metformin on specific single proteins in fetuses of HFD dams and fetuses of SD dams in the first overlap analysis. There was only one overlap (i.e., increased MRPS35) between the comparisons HFD-M vs. HFD and SD-M vs. SD (Figure 3a). Even when including all proteins with a *q*-value <0.1 and an fc ≥1.5-fold into the additional second overlap analysis, so as not to miss considerable effects due to the strict *q*-value cut off of <0.05, the number of overlaps increased by only two proteins (Figure 3b), which also were considerably altered by high fat diet (comparison HFD vs. SD). The two additional proteins were protein kinase C alpha (PRKCA) and ubiquitin-conjugating enzyme E2 E3 (UBE2D3). 

Next, surprisingly none of the 56 proteins significantly and relevantly altered by HFD was among the six proteins significantly altered by metformin under HFD (Figure 3a), suggesting an absence of compensation for high fat diet-induced alterations by metformin in fetal kidneys. However, looking further at the second overlap analysis of all proteins with a *q*-value <0.1 and an fc ≥1.5 (Figure 3b), we identified 11 proteins that showed considerable alterations induced both by HFD (comparison HFD vs. SD) and by metformin in HFD fetuses (comparison HFD-M vs. HFD). Remarkably, all these 11 proteins were counter-regulated by metformin, among them the interesting proteins ATP6V1G1, THY1, PRKCA and NDUFB3 (Table S2).

### 3.4. High Fat Diet During Mouse Pregnancy Significantly Affects the Levels of 56 Proteins in the Fetal Kidney at Term

To identify significant (*q* < 0.05) and relevant (fc ≥ 1.5-fold) alterations of single protein levels in fetal kidneys by the high fat diet, protein concentrations during the high fat diet without metformin (HFD group) were compared to concentrations during the standard diet without metformin (SD group). The levels of 56 proteins were significantly altered (Table 1). Forty-seven out of these proteins showed 3/3 valid values in the HFD group and 0/3 valid values in the SD group, and one protein showed 2/3 valid values in the HFD group and 0/3 valid values in the SD group. Therefore, forty-eight proteins were found to be increased in the HFD group compared to the SD group. One protein showed 3/3 valid values in both groups, and seven proteins showed 0/3 valid values in the HFD group and 3/3 valid values in the SD group. All these eight proteins were found to be significantly decreased in the HFD group. 

**Figure 4:**
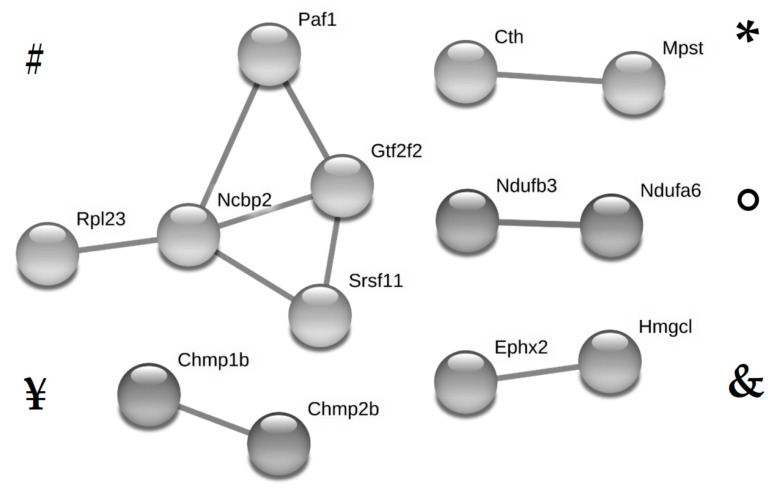
Clusters of potentially interacting, significantly (*q* < 0.05) and relevantly (fold change ≥1.5) altered proteins in fetal kidneys of dams fed a high fat diet without metformin (HFD group) compared to the group fed a standard diet without metformin (SD group) as identified by String analysis are shown. Symbols #, ¥, *, ◦, & were assigned to the clusters and correspond to the symbols in Table 1.

String analysis identified five clusters of potentially interacting proteins (Figure 4). The most prominent cluster of interacting proteins included proteins involved in processes of transcription/translation (RPL23, NCBP2, SRSF11, GTF2F2 and PAF1). Further predicted interactions were related to eicosanoid metabolism (EPHX2 and HMGCL), hydrogen sulfide H(2)S- synthesis (MPST and CTH), the respiratory chain of the mitochondrial membrane (NDUFB3, NDUFA6) and membrane remodeling (CHMP1B and CHMP2B). Uniprot Keywords identified were “acetylation” (24 proteins), “phosphoprotein” (35 proteins) and “mitochondrion” (12 proteins). The only biological process identified was “regulation of primary metabolic process” (29 proteins).

Literature research was performed for all proteins differentially affected by maternal high fat diet. Interestingly, known physiological relevance with regard to kidney disease was found primarily in proteins with high fold changes (SFN, AHSG, AMBP, ATP6V1G1, AQP1, THY1, LGALS9, SRI, DAPK1).

### 3.5. Metformin-Treatment in Healthy Pregnant Mice Significantly Alters the Levels of 29 Proteins in Fetal Kidneys at Term

To identify significant (*q* < 0.05) and relevant (fc ≥1.5-fold) alterations of single protein levels in fetal kidneys by metformin exposure in utero, protein levels during metformin treatment in standard diet animals (SD-M group) were compared to levels from untreated animals with a standard diet (SD group). The levels of 29 proteins were significantly altered (Table 2).

Twenty-seven of these proteins showed 3/3 valid values in the SD-M group and 0/3 valid values in the SD group and were thus found to be increased by metformin exposure in the standard diet. Two proteins were decreased by metformin exposure in the standard diet (0/3 valid values in the SD-M group and 3/3 valid values in the SD group).

**Figure 5:**
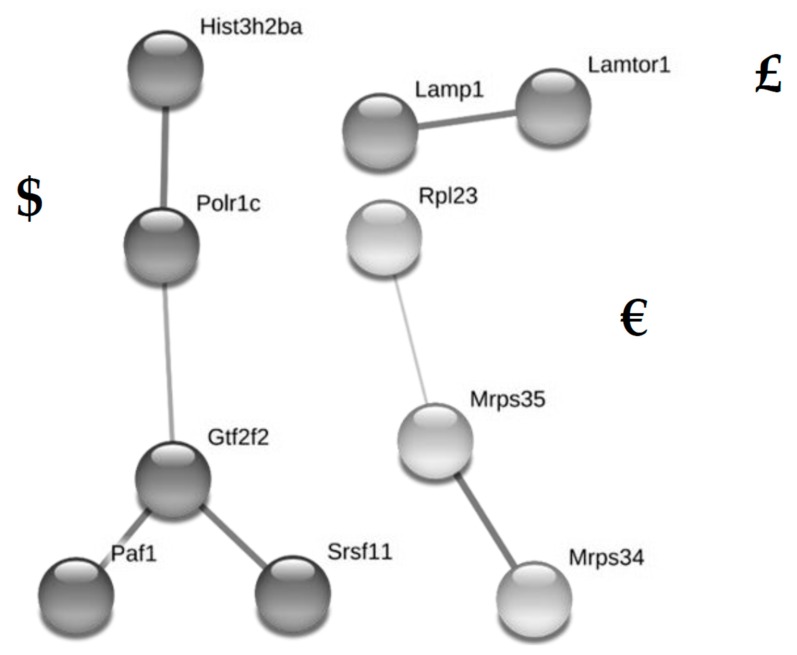
Clusters of potentially interacting, significantly (*q* < 0.05) and relevantly (fold change ≥1.5) altered proteins in fetal kidneys of dams fed the standard diet with metformin (SD-M group) compared to the group fed the standard diet without metformin (SD group) as identified by String analysis are shown. Symbols $, £, € were assigned to the clusters and correspond to the symbols in Table 2.

String analysis identified three clusters of potentially interacting proteins (Figure 5). The biggest cluster consisted of proteins related to transcription (HIST3H2BA, POLR1C, PAF1, SRSF11, GTF2F2); the second cluster of proteins was related to translation (RPL23, MRPS35, MRPS34); and the third interaction was shown for LAMP1 and LAMTOR1. Functional enrichment analysis of biological processes and Uniprot keywords yielded no results. 

Literature research indicated physiological relevance with regard to kidney disease for two proteins (AMBP, DAPK1). 

### 3.6. Metformin-Treatment in Obese Dams (Group HFD-M) Significantly Alters the Levels of Only Six Proteins in Fetal Kidneys Compared to Group HFD (High Fat Diet Without Metformin)

To identify significant (*q* < 0.05) and relevant (fc ≥1.5-fold) alterations of single protein levels in fetal kidneys by metformin treatment in obese dams, protein levels during metformin treatment in high fat diet animals (HFD-M group) were compared to levels from untreated animals with high fat diet (HFD group). The levels of only six proteins were significantly altered by this treatment (Table 3).

Looking at up- and downregulation, three proteins were increased by metformin treatment (3/3 valid values in the HFD-M group and 0/3 valid values in the HFD group) and three proteins were decreased by metformin treatment in obese dams. 

Due to the low number of altered proteins, String analysis could not identify a cluster of potentially interacting molecules. Similarly, functional enrichment analysis of biological processes and Uniprot keywords yielded no results.

Physiological relevance with regard to kidney disease as indicated by literature research was found for two proteins (PYCR1, FXYD2).

## 4. Discussion

Our study was designed to generate hypotheses on how maternal obesity and maternal metformin exposure might contribute to fetal programming of kidney disease. For this purpose, we studied the renal proteome of fetal mice close to term from four different experimental groups (standard diet +/− metformin treatment, high fat diet +/− metformin treatment). Interestingly, maternal high fat diet versus maternal standard diet had a larger effect on the proteome of fetal kidneys as estimated by the number of significantly and relevantly altered proteins (56 proteins affected) than metformin exposure in either standard diet mice (29 proteins affected) or high fat diet mice (6 proteins affected). Principal component analysis indicated a significant independent effect both of high fat diet and of metformin, the latter partially overcoming the diet effects and leading to similar global protein expression clusters, regardless of diet, before and during pregnancy. Both a high fat diet and metformin treatment in standard diet animals predominantly induced an increase rather than a decrease of affected proteins, but metformin during a high fat diet induced considerable counter-regulation of proteins dysregulated by a high fat diet. 

String analysis of proteins significantly altered by the maternal high fat diet identified the upregulation of several proteins potentially interacting in the process of transcription and translation (RPL23, NCBP2, SRS11, GTF2F2 and PAF1). This could be in line with the observation that most differentially affected proteins are increased by HFD rather than decreased. In addition, upregulation of proteins (CHMP1B and CHMP2B) involved in endosomal sorting required for transport complex III (ESCRT-III) might indicate increased membrane remodeling processes in HFD offspring [22]. The downregulation of NDUFB3 and NDUFA6 potentially interacting in the respiratory chain of the mitochondrial membrane, as well as altered levels of 11 other proteins categorized as “mitochondrial” by Uniprot Keyword Analysis, hints at a potential role of the mitochondria in the effect of HFD on renal programming. This is not surprising since mitochondrial damage significantly contributes to glomerulopathy and proximal tubular injury in high fat diet-induced kidney damage in adult mice [23]. However, our study shows for the first time that this process might start as early as in utero. 

In addition, String analysis could identify further interesting proteins which might contribute to fetal programming of kidney disease by maternal high fat diet. Thus, the upregulation of HMGCL and EPHX2 might lead to significant dysbalances of the eicosanoid metabolism. Cytochrome P450 eicosanoids like 20-hydroxyeicosatetraenoic acid (20-HETE) and epoxyeicosatrienoic acids are involved in the regulation of vascular tone, natriuresis and renal cyst formation [24]. Experimental inhibition of soluble epoxide hydrolase had blood pressure lowering effects in renovascular hypertension [25] and a gain-of-function EPHX2 polymorphism was shown to be associated with an increased incidence of acute kidney injury following cardiac surgery [26]. Mice deficient in soluble epoxide hydrolase were protected against streptozotocin-induced diabetic nephropathy [27]. Upregulation of two proteins related to hydrogen sulfide (H2S) synthesis might hint at a role of H2S in renal programming. In contrast to other gasotransmitters like nitric oxide (NO) and carbon monoxide (CO), the roles of H2S in kidney physiology are less well studied [28]. However, there is evidence that H2S has vasodilating [29] and anti-atherogenic properties [30].

Literature research (performed for all proteins significantly affected) further identified candidate proteins of special interest in the context of renal programming by HFD. Thus, 14-3-3 protein sigma (104.8-fold increase in HFD) is increased in many different kinds of renal pathology [31]. In addition, there is evidence that 14-3-3 protein sigma is involved in the activation of Akt/mTOR signaling by different stimuli [32,33]. Upregulation of alpha-2-HS-glycoprotein (35.4-fold), also known as fetuin-A, might especially be interesting in the context of vascular calcification, bone metabolism regulation and insulin resistance [34]. Protein AMBP (upregulation 32.1-fold) is expressed mainly in renal tubular cells and considered to be involved in the prevention of calcium oxalate crystal formation [35]. Upregulation of the catalytic subunit G1 of the V-type proton ATPase (ATP6V1G1; upregulated 14.3-fold) which is one of the enzymes that can cause distal renal acidosis [36], might alter renal concentration capacities or acid-base regulation by the kidney [37]. Aquaporin-1 upregulation (10.1-fold in our study) was lately shown to be protective against cyst development in models of autosomal dominant polycystic kidney disease [38]. On the other hand, upregulation of endothelial aquaporin-1 might be involved in the predisposition towards atherosclerosis via subendothelial intima compression [39]. For THY1 membrane glycoprotein (10-fold upregulated in our study), it was shown that overexpression decreases the activity of PPARγ and blocks adipocyte formation and it could therefore become a therapeutic target in obesity [40]. Galectin (upregulated 9.2-fold) was shown to be protective against Th1 and Th17 cell-mediated immune responses via enhanced Th2 immunity in the kidney [41]. In patients with diabetes type 2 and chronic kidney disease galectin is elevated [42]. Interestingly, galectin was able to suppress glomerular hypertrophy in db/db diabetic mice via cell-cycle regulatory mechanisms [43]. For protein kinase C alpha (PRKCA; -3.0-fold downregulated in our study), it was shown that downregulation may be protective in adult kidney disease [44,45], but disturbs nephron formation during kidney development in utero [46]. Finally, downregulation of DAPK1 (-48.6-fold) which is involved in pathways triggering apoptosis and autophagy might be protective against tubular injury from renal ischemia-reperfusion insults [47].

Comparable to the effect of high fat diet, metformin exposure in maternal standard diet induced the upregulation of proteins involved in transcription and translation. In maternal high fat diet, metformin exposure affected two proteins which are of putative interest in the context of kidney pathophysiology. Downregulation of PYCR1 (10.1-fold downregulated) might have anti-proliferative effects [48]. Downregulation of FXYD2 (-19.9 downregulated) might increase the activity of Na,K-ATPase, NCC and NKCC2 apical sodium transporters [49] which could in turn lead to sodium retention. However, FXYD2-knockout-mice were resistant to sodium retention and protected from salt-induced hypertension [50]. In humans, FXYD2 mutations can cause hypomagnesiaemia [51,52].

Surprisingly, in the first overlap analyses using a strict *q*-value cut off of <0.05, we did not identify any protein which was significantly altered by both the maternal high fat diet (HFD) compared to standard diet (SD) and, in the opposite direction, by metformin treatment in HFD animals. However, when looking at tendencies (*q*-value <0.1 instead of 0.05) still representing considerable alterations, we indeed found 11 proteins meeting the criteria of our second overlap analysis. Interestingly, all of them were regulated by HFD compared to SD in one direction and counter-regulated by metformin treatment in HFD animals compared to untreated HFD animals. Based on known physiological relevance in kidney pathophysiology as discussed above, ATP6V1G1, THY1, PRKCA and NDUFB3 represent the most promising candidates which might mediate “re-programming” effects of metformin therapy in maternal high fat diet. 

The absence of overlapping specific protein alterations by metformin in the high fat diet and standard diet groups despite similar global protein expression clusters in the principal component analysis may be surprising at first sight. However, the proteomes of fetal kidneys under the high fat diet compared to the standard diet clearly differ, so different regulatory events are needed to overcome the diet effects. 

Despite the clear design of the animal experiments and the interesting data, our study has a couple of limitations. First of all, our analysis did not consider the phosphorylation status of phospho-proteins. In addition, this study was designed as a hypothesis-generating study and did not include further analyses like western blots, histology or functional tests to confirm our results. Thus, the physiological relevance of our data remains to be elucidated by further studies.

In conclusion, our analyses provide evidence that both a maternal high fat diet and in-utero metformin exposure significantly impact upon the renal proteome of fetal male mice. With the help of String analysis and literature research we could identify interesting candidate proteins which might be involved in fetal programming of kidney disease. Beyond basic research, our results suggest that structured follow-up care with respect to early kidney disease should be discussed for all children born to obese mothers or mothers treated with metformin, e.g., renal ultrasound at birth and regular blood pressure measurements. 

## Figures and Tables

**Table 1:** Differentially expressed proteins (fc ≥ 1.5, *q* < 0.05) in kidneys of fetuses from dams fed a high fat diet without metformin (HFD group) compared to SD group fed a standard diet without metformin are shown.

Protein ID	Protein Name	*q*-value	Fold Change	Valid Values		Gene Name	Cluster	Functional Enrichments			
				SD	HFD						
O70456	14-3-3 protein sigma	<0.05	104.8	0	3	*Sfn*					
P62830	60S ribosomal protein L23	<0.001	65.0	0	3	*Rpl23*	#				
P43274	Histone H1.4	<0.05	49.7	0	3	*Hist1h1e*					
P29699	Alpha-2-HS-glycoprotein	<0.05	35.4	0	3	*Ahsg*					
Q07456	Protein AMBP	<0.05	32.1	0	3	*Ambp*					
E9Q8N1	Titin	<0.05	22.8	0	3	*Ttn*					
O55125	Protein NipSnap homolog 1	<0.05	16.5	0	3	*Nipsnap1*					
Q9CR51	V-type proton ATPase subunit G 1	<0.05	14.3	0	3	*Atp6v1g1*					
Q9D2U9	Histone H2B type 3-A	<0.001	12.0	0	3	*Hist3h2ba*					
Q02013	Aquaporin-1	<0.05	10.1	0	3	*Aqp1*					
A0A1L1SUX8	Thy-1 membrane glycoprotein	<0.05	10.0	0	3	*Thy1*					
B1AQR8	Galectin	<0.05	9.2	0	3	*Lgals9*					
Q9EP89	Serine beta-lactamase-like protein LACTB, mitochondrial	<0.05	9.1	0	3	*Lactb*					
P61028	Ras-related protein Rab-8B	<0.05	9.0	0	3	*Rab8b*					
Q8BJ64	Choline dehydrogenase, mitochondrial	<0.05	8.4	0	3	*Chdh*					
Q6P069	Sorcin	<0.05	8.0	0	3	*Sri*					
Q9D706	RNA polymerase II-associated protein 3	<0.05	7.2	0	3	*Rpap3*					
Q3UFY7	7-methylguanosine phosphate-specific 5-nucleotidase	<0.05	6.8	0	3	*Nt5c3b*					
P34914	Bifunctional epoxide hydrolase 2	<0.05	6.2	0	3	*Ephx2*	&				
Q3UW66	Sulfurtransferase	<0.05	6.1	0	3	*Mpst*	*				
Q3ULB1	Testin	<0.05	6.0	0	3	*Tes*					
Q8BJF9	Charged multivesicular body protein 2b	<0.05	5.8	0	3	*Chmp2b*	¥				
J3QJX3	Protein sel-1 homolog 1	<0.05	5.6	0	3	*Sel1l*					
Q3TE40	Replication protein A 32 kDa subunit	<0.001	5.4	0	3	*Rpa2*					
Q8K2T8	RNA polymerase II-associated factor 1 homolog	<0.05	5.0	0	3	*Paf1*	#				
D3Z3D2	Nuclear cap-binding protein subunit 2	<0.05	4.9	0	3	*Ncbp2*	#				
Q99LU0	Charged multivesicular body protein 1b-1	<0.05	4.6	0	3	*Chmp1b1*	¥				
Q9CZR8	Elongation factor Ts, mitochondrial	<0.05	4.6	0	3	*Tsfm*					
Q8R409	Protein HEXIM1	<0.05	4.4	0	3	*Hexim1*					
Q8BGY7	Protein FAM210A	<0.05	4.3	0	3	*Fam210a*					
O88665	Bromodomain-containing protein 7	<0.001	4.3	0	3	*Brd7*					
Q3UIX4	N/A	<0.05	4.1	0	3	*Srsf11*	#				
E9QMC1	Cingulin	<0.05	4.0	0	3	*Cgn*					
A0A0R4J1N9	Transcription factor A, mitochondrial	<0.05	4.0	0	3	*Tfam*					
Q9JIK9	28S ribosomal protein S34, mitochondrial	<0.05	3.9	0	3	*Mrps34*					
A0A1L1SSA8	Transmembrane protein 205	<0.05	3.9	0	3	*Tmem205*					
Q99LS3	Phosphoserine phosphatase	<0.01	3.9	0	3	*Psph*					
Q8BHS6	Armadillo repeat-containing X-linked protein 3	<0.05	3.8	0	3	*Armcx3*					
Q8R0A0	General transcription factor IIF subunit 2	<0.05	3.6	0	3	*Gtf2f2*	#				
O89051	Integral membrane protein 2B	<0.05	3.4	0	3	*Itm2b*					
Q8VCN5	Cystathionine gamma-lyase	<0.05	3.3	0	3	*Cth*	*				
O35682	Myeloid-associated differentiation marker	<0.05	3.3	0	3	*Myadm*					
Q3UI43	BRISC and BRCA1-A complex member 1	<0.05	3.2	0	3	*Babam1*					
P38060	Hydroxymethylglutaryl-CoA lyase, mitochondrial	<0.001	3.1	0	2	*Hmgcl*	&				
O55106	Striatin	<0.05	3.0	0	3	*Strn*					
A0A140LIQ1	Transforming acidic coiled-coil-containing protein 2	<0.05	2.9	0	3	*Tacc2*					
Q3UE37	Ubiquitin-conjugating enzyme E2 Z	<0.05	2.4	0	3	*Ube2z*					
Q9WVL0	Maleylacetoacetate isomerase	<0.05	1.8	0	3	*Gstz1*					
Q9ESU7	Amino acid transporter	<0.05	−1.5	3	3	*Slc1a5*					
E9PZY8	Protein virilizer homolog	<0.05	−2.1	3	0	*Virma*					
Q4VA93	Protein kinase C	<0.05	−3.0	3	0	*Prkca*					
Q9CQZ6	NADH dehydrogenase [ubiquinone] 1 beta subcomplex subunit 3	<0.001	−9.6	3	0	*Ndufb3*	°				
Q9CQZ5	NADH dehydrogenase [ubiquinone] 1 alpha subcomplex subunit 6	<0.05	−10.5	3	0	*Ndufa6*	°				
D3YZN4	Paraplegin	<0.001	−12.3	3	0	*Spg7*					
E9QAE4	Histone-lysine N-methyltransferase	<0.05	−17.8	3	0	*Nsd1*					
Q80YE7	Death-associated protein kinase 1	<0.001	−48.6	3	0	*Dapk1*					

SD, standard diet without metformin; HFD, high fat diet without metformin; fold change: positive values indicate upregulation in HFD compared to SD, negative values indicate downregulation; N/A, not available. Color codes indicate functional enrichments (FDR < 0.01) identified by STRING (red marks, Phosphoprotein, orange marks, Regulation of primary metabolic process, blue marks, Acetylation, yellow marks, Mitochondrion). Symbols (#, ¥, *, ◦, &) indicate affiliation with different clusters identified by STRING analysis (see Figure 4).

**Table 2:** Differentially expressed proteins (fc ≥ 1.5, *q* < 0.05) in kidneys of fetuses from dams fed a standard diet with metformin (SD-M group) compared to SD group fed a standard diet without metformin are shown.

Protein ID	Protein Name	*q*-value	Fold Change	Valid Values		Gene Name	Cluster
				SD	SD-M		
P62830	60S ribosomal protein L23	<0.05	106.8	0	3	*Rpl23*	€
Q07456	Protein AMBP	<0.05	31.4	0	3	*Ambp*	
O55125	Protein NipSnap homolog 1	<0.001	16.6	0	3	*Nipsnap1*	
P11438	Lysosome-associated membrane glycoprotein 1	<0.05	15.5	0	3	*Lamp1*	£
Q9D2U9	Histone H2B type 3-A	<0.05	13.6	0	3	*Hist3h2ba*	$
G3UXX5	DNA-directed RNA polymerases I and III subunit RPAC1	<0.05	11.3	0	3	*Polr1c*	$
Q9EP89	Serine beta-lactamase-like protein LACTB, mitochondrial	<0.05	9.0	0	3	*Lactb*	
Q9D706	RNA polymerase II-associated protein 3	<0.05	8.2	0	3	*Rpap3*	
Q8K2T8	RNA polymerase II-associated factor 1 homolog	<0.05	7.9	0	3	*Paf1*	$
Q3ULB1	Testin	<0.05	6.1	0	3	*Tes*	
O88665	Bromodomain-containing protein 7	<0.05	5.3	0	3	*Brd7*	
Q9JIK9	28S ribosomal protein S34, mitochondrial	<0.05	5.1	0	3	*Mrps34*	€
P11157	Ribonucleoside-diphosphate reductase subunit M2	<0.05	5.0	0	3	*Rrm2*	
Q3UIX4	Serine/arginine-rich-splicing factor 11	<0.05	5.0	0	3	*Srsf11*	$
A0A0R4J0L6	28S ribosomal protein S35, mitochondrial	<0.05	5.0	0	3	*Mrps35*	€
Q9DBX2	Phosducin-like protein	<0.01	4.8	0	3	*Pdcl*	
H7BX44	Non-specific serine/threonine protein kinase	<0.05	4.7	0	3	*Cdc42bpa*	
Q8R0A0	General transcription factor IIF subunit 2	<0.05	4.2	0	3	*Gtf2f2*	$
F8WIK0	Anamorsin	<0.05	4.2	0	3	*Ciapin1*	
E9QMC1	Cingulin	<0.05	3.7	0	3	*Cgn*	
O55106	Striatin	<0.01	3.5	0	3	*Strn*	
O89051	Integral membrane protein 2B	<0.001	3.4	0	3	*Itm2b*	
Q8N7N5	DDB1- and CUL4-associated factor 8	<0.05	2.8	0	3	*Dcaf8*	
Q3UH93	Plexin-D1	<0.05	2.7	0	3	*Plxnd1*	
A0A0R4J0G4	Ran-binding protein 10	<0.05	2.6	0	3	*Ranbp10*	
P52483	Ubiquitin-conjugating enzyme E2 E3	<0.05	2.3	0	3	*Ube2e3*	
G3UZ63	Kelch domain-containing protein 4	<0.05	2.3	0	3	*Klhdc4*	
A0A0A6YX02	Ragulator complex protein LAMTOR1	<0.05	−10.3	3	0	*Lamtor1*	£
Q80YE7	Death-associated protein kinase 1	<0.05	−48.7	3	0	*Dapk1*	

SD, standard diet without metformin; SD-M, standard diet with metformin; fold change: positive values indicate upregulation in SD-M compared to SD, negative values indicate downregulation; Symbols ($, £, €) indicate affiliation with different clusters identified by STRING analysis (see Figure 5).

**Table 3:** Differentially expressed proteins (fc ≥1.5, *q* < 0.05) in kidneys of fetuses from dams fed a high fat diet with metformin (HFD-M group) compared to HFD group fed a high fat diet without metformin are shown.

Protein ID	Protein Name	*q*-value	Fold Change	Valid Values		Gene Name
				HFD	HFD-M	
A0A0R4J0L6	28S ribosomal protein S35, mitochondrial	<0.05	8.6	0	3	*Mrps35*
Q8K2C9	Very-long-chain (3R)-3-hydroxyacyl-CoA dehydratase 3	<0.05	7.4	0	3	*Hacd3*
E9Q784	Zinc finger CCCH domain-containing protein 13	<0.001	5.7	0	3	*Zc3h13*
Q9Z1J3	Cysteine desulfurase, mitochondrial	<0.05	−3.0	3	0	*Nfs1*
Q922W5	Pyrroline-5-carboxylate reductase 1, mitochondrial	<0.05	−10.1	3	0	*Pycr1*
Q04646	Sodium/potassium-transporting ATPase subunit gamma	<0.05	−19.9	3	0	*Fxyd2*

HFD, high fat diet without metformin; HFD-M, high fat diet with metformin; fold change: positive values indicate upregulation in HFD-M compared to HFD, negative values indicate downregulation.

## Supplementary Materials

The following are available online at https://www.mdpi.com/2077-0383/8/5/663/s1: Figure S1: Overlap cluster of three proteins significantly and relevantly (*q* < 0.05, fc ≥ 1.5) altered in fetal kidneys both by high fat diet (HFD compared to SD group) and by metformin during standard diet (SD-M compared to SD group) as identified by String analysis is shown. Srsf11, 60S ribosomal protein L23; Gtf2f2, Cingulin; Paf1, Integral membrane protein 2B, Table S1: Overlaps of proteins significantly and relevantly (*q* < 0.05, fc ≥ 1.5) altered in fetal kidneys both by high fat diet (HFD compared to SD group) and by metformin during standard diet (SD-M compared to SD group) are shown, Table S2: Overlaps of proteins considerably and relevantly altered (*q* < 0.1, fc ≥ 1.5) in fetal kidneys both by high fat diet (HFD compared to SD group) and by metformin during high fat diet (HFD-M compared to HFD group) are shown.
