# Supplementary material for: Maternal High Fat Diet and in-Utero Metformin Exposure Significantly Impact upon the Fetal Renal Proteome of Male Mice
Source: J Clin Med. 2019 May 11;8(5):663. doi: 10.3390/jcm8050663 (PMC6571731; doi:10.3390/jcm8050663)
Supplement: Supplementary file 1 [file jcm-08-00663-s001.pdf]

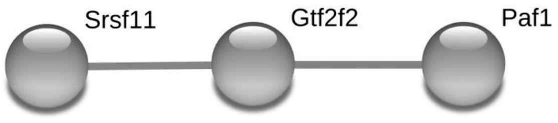

Figure S1. Overlap cluster of three proteins significantly and relevantly ($q < 0.05$, fc ≥ 1.5) altered in fetal kidneys both by high fat diet (HFD compared to SD group) and by metformin during standard diet (SD-M compared to SD group) as identified by String analysis is shown. Srsf11, 60S ribosomal protein L23; Gtf2f2, Cingulin; Paf1, Integral membrane protein 2B.

Table S1. Overlaps of proteins significantly and relevantly ($q < 0.05$, $fc \geq 1.5$) altered in fetal kidneys both by high fat diet (HFD compared to SD group) and by metformin during standard diet (SD-M compared to SD group) are shown.

Protein ID	Protein name	q-value		Fold change		Gene name
		HFD vs SD	SD-M vs SD	HFD vs SD	SD-M vs SD	
P62830	60S ribosomal protein L23	<0.001	<0.05	65.0	106.8	Rpl23
Q07456	Protein AMBP	<0.05	<0.05	32.1	31.4	Ambp
O55125	Protein NipSnap homolog 1	<0.05	<0.001	16.5	16.6	Nipsnap1
Q9D2U9	Histone H2B type 3-A	<0.001	<0.05	12.0	13.6	Hist3h2ba
Q9EP89	Serine beta-lactamase-like protein LACTB, mitochondrial	<0.05	<0.05	9.1	9.0	Lactb
Q9D706	RNA polymerase II-associated protein 3	<0.05	<0.05	7.2	8.2	Rpap3
Q8K2T8	RNA polymerase II-associated factor 1 homolog	<0.05	<0.05	5.0	7.9	Paf1
Q3ULB1	Testin	<0.05	<0.05	6.0	6.1	Tes
O88665	Bromodomain-containing protein 7	<0.001	<0.05	4.3	5.3	Brd7
Q9JIK9	28S ribosomal protein S34, mitochondrial	<0.05	<0.05	3.9	5.1	Mrps34
Q3UIX4	Serine and arginine rich splicing factor 11	<0.05	<0.05	4.1	5.0	Srsf11
Q8R0A0	General transcription factor IIF subunit 2	<0.05	<0.05	3.6	4.2	Gtf2f2
E9QMC1	Cingulin	<0.05	<0.05	4.0	3.7	Cgn
O55106	Striatin	<0.05	<0.05	3.0	3.5	Strn
O89051	Integral membrane protein 2B	<0.05	<0.001	3.4	3.4	Itm2b
Q80YE7	Death-associated protein kinase 1	<0.001	<0.05	-48.7	-48.7	Dapk1

HFD, high fat diet without metformin; SD, standard diet without metformin; SD-M, standard diet with metformin; vs, compared to; fc, fold change: positive values indicate up-regulation in the respective group comparisons, negative values indicate down-regulation.

Table S2. Overlaps of proteins considerably and relevantly altered (q < 0.1, fc ≥ 1.5) in fetal kidneys both by high fat diet (HFD compared to SD group) and by metformin during high fat diet (HFD-M compared to HFD group) are shown.

Protein ID	Protein name	q-value		Fold change		Gene name
		HFD vs SD	HFD-M vs HFD	HFD vs SD	HFD-M vs HFD	
K3W4R2	Myosin-14	=0.05	<0.1	15.7	-15.4	Myh14
Q9CR51	V-type proton ATPase subunit G 1	<0.05	<0.1	14.3	-14.6	Atp6v1g1
A0A0G2JE32	Ubiquitin-conjugating enzyme E2 D3	<0.1	<0.1	13.0	-18.0	Ube2d3
A0A1L1SUX8	Thy-1 membrane glycoprotein	<0.05	<0.1	10.0	-9.0	Thy1
Q922W5	Pyrroline-5-carboxylate reductase 1, mitochondrial	<0.1	=0.05	6.0	-10.1	Pycr1
P28667	MARCKS-related protein	<0.1	<0.1	5.3	-9.4	Marcksl1
Q3UI43	BRISC and BRCA1-A complex member 1	=0.05	<0.1	3.2	-3.2	Babam1
E9PZY8	Protein virilizer homolog	<0.05	<0.1	-2.1	1.9	1110037F02 Rik
Q4VA93	Protein kinase C	<0.05	<0.1	-3.0	2.6	Prkca
Q9CQZ6	NADH dehydrogenase [ubiquinone] 1 beta subcomplex subunit 3	<0.001	<0.1	-9.6	9.1	Ndufb3
D3YZN4	Paraplegin	<0.001	<0.1	-12.3	14.0	Spg7

HFD, high fat diet without metformin; SD, standard diet without metformin; SD-M, standard diet with metformin; vs, compared to; fc, fold change: positive values indicate up-regulation in the respective group comparisons, negative values indicate down-regulation.
